# Multicomponent Intervention to Improve Acute Myocardial Infarction Care in Tanzania: Protocol for a Pilot Implementation Trial

**DOI:** 10.2196/59917

**Published:** 2024-09-24

**Authors:** Julian T Hertz, Francis M Sakita, Faraan O Rahim, Blandina T Mmbaga, Frida Shayo, Vivian Kaboigora, Julius Mtui, Gerald S Bloomfield, Hayden B Bosworth, Janet P Bettger, Nathan M Thielman

**Affiliations:** 1 Duke Global Health Institute Duke University Durham, NC United States; 2 Department of Emergency Medicine Duke University School of Medicine Durham, NC United States; 3 Kilimanjaro Christian Medical Centre Moshi United Republic of Tanzania; 4 Department of Internal Medicine Duke University School of Medicine Durham, NC United States; 5 Department of Population Health Sciences Duke University School of Medicine Durham, NC United States

**Keywords:** myocardial infarction, Tanzania, sub-Saharan Africa, implementation science, quality improvement

## Abstract

**Background:**

Although the incidence of acute myocardial infarction (AMI) is rising in sub-Saharan Africa, the uptake of evidence-based care for the diagnosis and treatment of AMI is limited throughout the region. In Tanzania, studies have revealed common misdiagnosis of AMI, infrequent administration of aspirin, and high short-term mortality rates following AMI.

**Objective:**

This study aims to evaluate the implementation and efficacy outcomes of an intervention, the Multicomponent Intervention to Improve Acute Myocardial Infarction Care (MIMIC), which was developed to improve the delivery of evidence-based AMI care in Tanzania.

**Methods:**

This single-arm pilot trial will be conducted in the emergency department (ED) at a referral hospital in northern Tanzania. The MIMIC intervention will be implemented by the ED staff for 1 year. Approximately 400 adults presenting to the ED with possible AMI symptoms will be enrolled, and research assistants will observe their care. Thirty days later, a follow-up survey will be administered to assess mortality and medication use. The primary outcome will be the acceptability of the MIMIC intervention, which will be measured by the Acceptability of Intervention Measurement (AIM) instrument. Acceptability will further be assessed via in-depth interviews with key stakeholders. Secondary implementation outcomes will include feasibility and fidelity. Secondary efficacy outcomes will include the following: the proportion of participants who receive electrocardiogram and cardiac biomarker testing, the proportion of participants with AMI who receive aspirin, 30-day mortality among participants with AMI, and the proportion of participants with AMI taking aspirin 30 days following enrollment.

**Results:**

Implementation of MIMIC began on September 1, 2023. Enrollment is expected to be completed by September 1, 2024, and the first results are expected to be published by December 31, 2024.

**Conclusions:**

This study will be the first to evaluate an intervention for improving AMI care in sub-Saharan Africa. If MIMIC is found to be acceptable, the findings from this study will inform a future cluster-randomized trial to assess effectiveness and scalability.

**Trial Registration:**

ClinicalTrials.gov NCT04563546; https://clinicaltrials.gov/study/NCT04563546

**International Registered Report Identifier (IRRID):**

DERR1-10.2196/59917

## Introduction

### Overview

Acute myocardial infarction (AMI) is a leading cause of death and disability globally [1]. In sub-Saharan Africa (SSA), the burden of AMI is rising rapidly as the region progresses through its epidemiological transition [2]. Approximately 250,000 deaths, or 5% of all deaths in SSA each year, are attributed to AMI [3]. In an effort to curb the rising global burden of AMI, the World Health Organization has prioritized evidence-based AMI treatment with antiplatelet agents as a “Best Buy” for reducing noncommunicable disease morbidity and mortality [4]. Yet, AMI care remains under-studied in SSA [2,5-7], and there are currently no published studies from the region evaluating quality improvement interventions to increase aspirin administration in AMI [8,9]. In Tanzania, recent studies have shown that uptake of evidence-based AMI care is suboptimal. Specifically, in a northern Tanzanian emergency department (ED), we found that only half of adult ED patients with potential AMI symptoms undergo testing with electrocardiogram (ECG) or cardiac biomarker testing, only 23% of patients with AMI were treated with aspirin, and only 5% of patients with AMI were taking aspirin 30 days following their diagnosis [10-12]. Against this backdrop, AMI outcomes in Tanzania are currently poor: approximately 90% of AMI cases are misdiagnosed, and 30-day mortality among patients with AMI is 43% [12].

In response to these findings, an interdisciplinary design team consisting of providers from the study hospital and global experts in implementation science, emergency medicine, and cardiology, developed a multicomponent intervention to improve the uptake of evidence-based AMI care in Tanzania. Because there are currently no published interventions to improve AMI care in SSA [8], the design team chose to adopt a quality improvement intervention from Brazil (the ACS-BRIDGE intervention) [13,14]. The team chose this intervention due to its emphasis on both diagnosis and treatment, its multicomponent nature, its appropriateness for a resource-limited setting, its preliminary efficacy, and its congruence with local barriers to care [15]. Guided by formative qualitative data [15], the design team tailored the ACS-BRIDGE intervention to the local Tanzanian context using the ADAPT-ITT framework [16]. The result was the tailored intervention, Multicomponent Intervention to improve acute Myocardial Infarction Care (MIMIC). This pilot trial will assess the implementation outcomes of MIMIC as well as its impact on clinical care in a Tanzanian ED.

### Study Aims and Outcomes

This study will assess the acceptability, feasibility, and preliminary efficacy of the MIMIC intervention in a single-arm pilot trial. The primary outcome will be the acceptability of the MIMIC intervention. Secondary implementation outcomes will include feasibility and fidelity; secondary efficacy outcomes will include (1) the proportion of participants with chest pain or shortness of breath who undergo diagnostic testing for AMI, (2) the proportion of AMI participants who receive aspirin, (3) 30-day mortality among AMI participants, and (4) the proportion of AMI participants adhering to aspirin at 30 days following study enrollment.

## Methods

### Adaptation

The process of developing the MIMIC intervention has been described in detail elsewhere [17]. Briefly, an interdisciplinary design team considered a wide range of published interventions to improve AMI care [8,13,18-25]. Two existing quality improvement interventions for AMI clinical care were considered most suitable for potential adaptation to the resource-limited Tanzanian health care context. In India, the ACS-QUIK trial aimed to improve AMI care, but did not demonstrate a significant reduction in mortality or cardiovascular events among patients, included strategies (such as electronic discharge order sets) that were not possible in Tanzania, and emphasized rapid percutaneous coronary intervention, which is not currently available in Tanzania [21]. For these reasons, the ACS-QUIK study was not selected for adaptation. The other intervention, ACS-BRIDGE in Brazil, successfully bolstered adherence to evidence-based AMI therapies, reduced in-hospital major cardiovascular events, and included low-cost strategies that were more feasible in Tanzania, so it was selected for adaptation [13]. However, the health care landscape in Tanzania is much different from Brazil, with fewer resources and less access to advanced cardiac care [26]. To address this, the MIMIC intervention was adapted to the resource-limited context of Tanzania over a 1-year period of discussion and refinement by an international team of experts in emergency medicine, cardiology, and implementation science [17].

### Design

**Figure 1:**
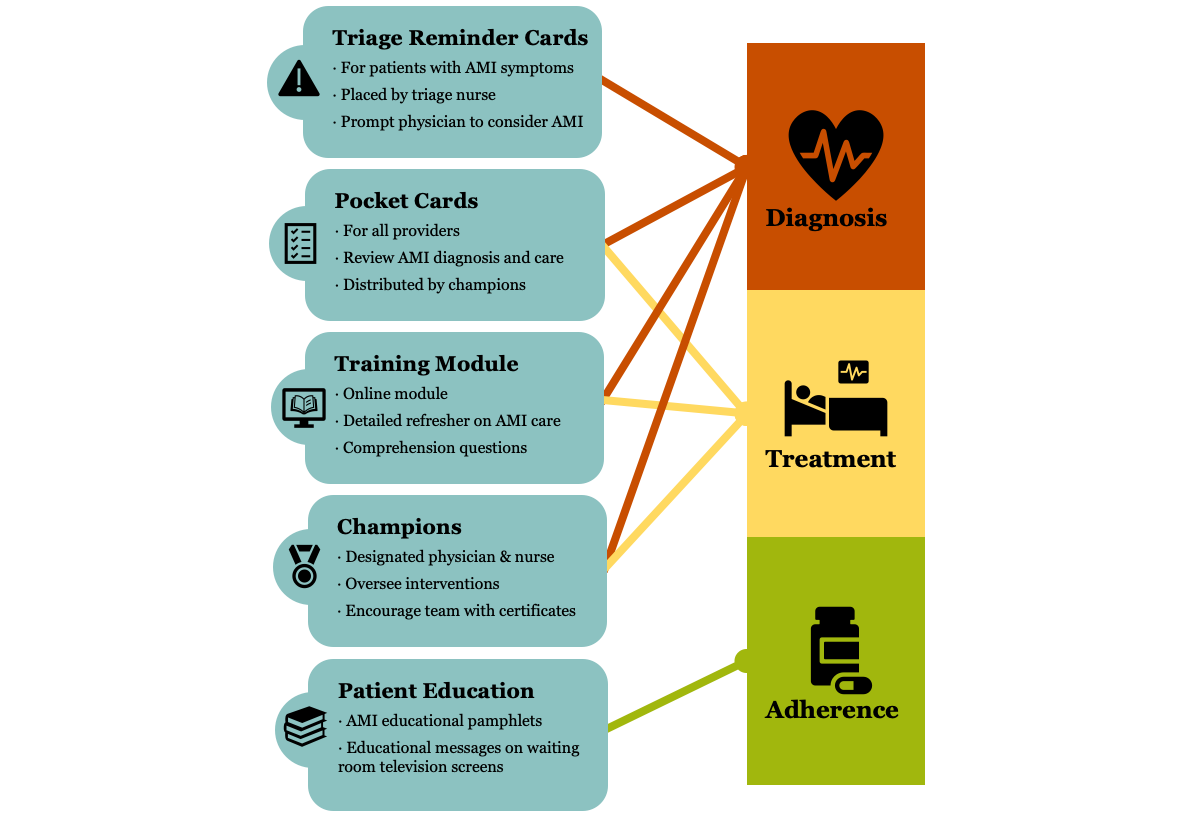
The MIMIC intervention for improving acute myocardial infarction care in Tanzania. AIM: acceptability of intervention measure; AMI: acute myocardial infarction; MIMIC: multicomponent intervention to improve acute myocardial infarction care.

The MIMIC intervention is delineated in Figure 1. Briefly, the intervention consists of five key components: (1) a triage card that will be placed on the stretchers of patients with AMI symptoms by the triage nurse to prompt the physician to consider the diagnosis of AMI, (2) a pocket card summarizing the basic approach to AMI diagnosis and care that will be distributed to physicians and nurses, (3) a detailed web-based refresher module covering AMI diagnosis and care that will be required for all staff physicians and nurses, (4) educational pamphlets that will be distributed to patients with AMI and educational messages regarding AMI self-care that will be displayed on screens in the ED waiting room, and (5) a designated physician and nurse champion who will encourage the team and manage the delivery of all 5 intervention components. The full MIMIC intervention has been previously published. As described elsewhere, the patient education materials were piloted with patients from a range of educational backgrounds to ensure comprehensibility during the intervention development process [17].

### Study Setting

The study will be conducted at Kilimanjaro Christian Medical Centre (KCMC), a tertiary care center in Moshi, Tanzania. KCMC is a zonal hospital covering a catchment area of approximately 15 million persons and was the site of our preliminary studies identifying gaps in evidence-based AMI care [10-12,15,27]. KCMC is currently equipped with basic AMI diagnostic tools, including ECG machines, both point-of-care and laboratory-based troponin assays, and echocardiograms. KCMC is also currently well-stocked with basic AMI medications, including aspirin, clopidogrel, heparin, statins, nitrates, beta-blockers, other antihypertensives, and thrombolytics. Additionally, KCMC offers a dedicated outpatient follow-up clinic for patients with cardiac conditions like ischemic heart disease. However, KCMC presently does not have a cardiologist on staff and lacks the capacity for percutaneous coronary intervention or coronary surgery.

### Study Sample

This pilot trial will be conducted in the KCMC ED for 1 year. Implementation outcomes (acceptability, feasibility, and sustainability) will be assessed primarily via in-depth interviews and surveys conducted with key stakeholders (physicians, nurses, patients, and administrators). Secondary efficacy outcomes will be assessed via observation of the care of patients who present to the KCMC ED with possible AMI symptoms during the study period. Based on the results of prior surveillance studies in the KCMC ED [11,12,28], we expect to enroll approximately 400 patients with chest pain or dyspnea in the KCMC ED over 1 year.

### Inclusion and Exclusion Criteria

For in-depth interviews and surveys assessing implementation outcomes, eligible participants will be KCMC staff, including hospital staff and physicians and nurses working in the ED during the study period, as well as patients diagnosed with AMI in the KCMC ED during the study period. Patients younger than 18 years and those who were unable to provide informed consent were excluded from study participation. For ED patients whose care will be observed to assess secondary efficacy outcomes, eligibility criteria will be as follows: (1) KCMC ED patient presenting during the study period, (2) being 18 years or older, and (3) primary or secondary complaint of chest pain or dyspnea. Exclusion criteria will be as follows: (1) chest pain secondary to trauma, (2) self-reported fever, and (3) inability to provide informed consent. Consistent with prior AMI studies in Tanzania [11,12,28], these exclusion criteria were selected to exclude patients whose chest pain or dyspnea is unlikely to be due to AMI.

### Recruitment

For surveys administered to hospital staff to assess implementation outcomes, members of the research team will approach physicians, nurses, and administrators to offer them participation in the study. To minimize disruptions to clinical care, research staff will approach staff during break periods, meals, or at the conclusion of their shift. Staff will be compensated 5000 Tanzanian shillings (approximately US $2) for their time completing the survey.

For in-depth interviews evaluating implementation outcomes, purposive sampling will be used to ensure participants from diverse backgrounds are included. Participants will be hospital staff, administrators, and patients from a variety of occupations, ages, genders, and educational backgrounds. Selected hospital staff and administrators will again be approached by research staff during their break periods and offered participation in the in-depth interviews. Moreover, selected patients will be contacted via telephone and offered participation. All in-depth interview participants will be compensated for their time (approximately US $2).

### Procedures

#### MIMIC Implementation

The MIMIC intervention will be implemented by the clinical staff, not members of the research team. Following the initiation of MIMIC, the continuum of care received by ED patients with AMI will be directed by all 5 components of the intervention. Triage nurses will identify patients presenting to the ED with chest pain, dyspnea, or other possible AMI symptoms and will affix “ACS Suspect” cards to their stretchers. A list of possible AMI symptoms will be posted in the triage area to aid triage nurses in the identification of patients with possible AMI. The “ACS Suspect” cards serve as a notification to ED physicians, prompting them to consider the possibility of AMI and follow treatment protocols for the condition. ED physicians will also carry pocket cards that provide an overview of AMI diagnosis and serve as an accessible reference for treatment procedures. After being diagnosed with AMI, patients will also be provided an educational pamphlet that provides health literacy resources for AMI and cardiovascular disease prevention, including information on healthy diet, exercise, and medication adherence. In the event a patient is too ill to receive the pamphlet directly, the pamphlet will be given to a relative. All KCMC ED physicians and nurses will also be required to complete a web-based module providing an in-depth review of evidence-based AMI diagnosis and treatment. Staff will only be required to complete the module once, although they will continue to have access to the web-based module if they wish to return to it. Designated physician and nurse “champions” will also encourage team members to improve AMI care at daily staff meetings, ensure implementation of all intervention components, and recognize staff who provide outstanding AMI care with congratulatory certificates.

#### Provider Surveys

To assess acceptability and other implementation outcomes, trained research assistants will approach ED staff to administer provider surveys (Multimedia Appendix 1). These provider surveys will be administered beginning 1 month after intervention implementation, and survey administration will continue over the course of the trial period. Surveys will use the 4-item acceptability of intervention measure (AIM) [29] to evaluate provider perceptions of the acceptability of the MIMIC intervention. The survey will also include the 4-item Feasibility of Intervention Measure (FIM) [29] to evaluate feasibility. Additional survey questions will elicit provider opinions regarding specific components of the MIMIC intervention. Members of the research team will administer provider surveys to ED staff over the course of the pilot trial, with the goal of administering the survey to at least 90% of staff by the trial’s conclusion.

#### In-Depth Interviews

After initiation of the MIMIC intervention, research assistants will conduct in-depth interviews with providers, patients, and administrators to obtain additional qualitative data regarding the acceptability of MIMIC and other implementation outcomes. Interviews will be guided by a semistructured interview guide, informed by the Theoretical Framework for Acceptability, which encompasses 7 domains of acceptability: affective attitude, burden, perceived effectiveness, ethicality, intervention coherence, opportunity costs, and self-efficacy [30,31]. In-depth interviews will also explore stakeholder perceptions of the feasibility of the MIMIC intervention. Interviews will be conducted face-to-face in Swahili and will last approximately 1 hour. Interviews will be conducted until thematic saturation is achieved [32]. The epistemology informing these qualitative interviews is constructivist, recognizing that knowledge is constructed through the unique, lived experiences of providers, patients, and hospital administrators, in addition to the social interactions amongst them [33].

#### Observation of Intervention Fidelity

Trained research assistants will observe KCMC ED clinical care from 8 AM until 11 PM seven days per week during the trial period. They will observe and record the number of nurses who are using the “ACS Suspect” triage card and the number of physicians who have their pocket cards. Research staff will also monitor the web-based training module and record the total number of staff who complete the training during the trial period. Additionally, research staff will also observe the uptake of the patient educational pamphlets and triage cards, as described below.

#### Observation of Clinical Care

For 1 year following the initiation of MIMIC (September 1, 2023, to September 1, 2024), research assistants will enroll adult patients (age ≥18 years) presenting to the KCMC ED with a primary or secondary complaint of chest pain or shortness of breath. Written, informed consent will be obtained from all patients interested in participating. Following enrollment, a comprehensive approach will be taken to observe relevant testing and treatment procedures administered to the participants. Research staff will observe and record whether patients had an “ACS Suspect” triage card affixed to their stretcher and whether cardiac biomarkers and ECGs were obtained by the ED clinicians. The findings of all ED investigations, including cardiac biomarkers and ECGs when obtained, will be recorded. Additionally, physician-documented diagnoses will be transcribed directly from patients’ electronic medical records. Research staff will also observe and record all ED treatments, including aspirin administration, and will note whether the patient received an educational pamphlet.

Enrolled patients will be contacted 30 days following enrollment to assess mortality and medication use. Medication use at 30 days will be determined by participant self-report. The 4-item AIM instrument [29] will also be administered to participants who received the educational pamphlet to discern patient perspectives on the acceptability of this component of the MIMIC intervention. Trained research assistants will conduct these 30-day follow-up assessments via telephone; when participants are unreachable by phone, a member of the study team will visit their home to administer the follow-up survey in person.

### Implementation Outcomes

#### Primary Outcome: Acceptability

Implementation outcomes are summarized in Table 1. The primary outcome of this pilot trial will be the acceptability of the MIMIC intervention. Acceptability will be measured by the AIM instrument [29], embedded within the provider survey. The AIM instrument measures acceptability as an implementation outcome of an intervention, and it has shown substantial validity through rigorous psychometric assessment [29]. The AIM instrument is administered in survey format to respondents. Responses to each question are on a 5-point Likert Scale (Strongly Agree=5, Agree=4, Neutral=3, Disagree=2, Strongly Disagree=1). Responses of “Strongly Agree” (5) and “Agree” (4) are considered to indicate a more acceptable intervention. The responses to each question will be averaged to give an acceptability score between 1 and 5 for each respondent. The primary outcome will be the overall mean acceptability score among respondents. The AIM instrument does not have a validated cutoff score, although higher scores indicate greater acceptability [29]. Consistent with the approach of other studies that have used the AIM instrument, a mean acceptability score ≥ 4 will be considered indicative of acceptability for our study [34-42]. Acceptability will also be assessed via the qualitative data obtained from the in-depth interviews with key stakeholders, as described above.

**Table 1:** Implementation outcomes and measures.

Implementation outcomes [43]	Measures
Acceptability^a^	Mean acceptability score on AIM^b^ instrument administered via ED^c^ provider surveys Qualitative data obtained from in-depth interviews with hospital staff, administrators, and patients
Feasibility	Mean feasibility score on FIM instrument administered via ED provider surveys Total number of triage cards and pocket cards distributed and replaced Number of ED providers unable to complete web-based module and average minutes required to complete training module
Fidelity	Proportion of enrolled patients with chest pain or shortness of breath who are flagged with the red triage card, as determined by research staff direct observation. Proportion of ED physicians receiving pocket cards and starting web-based training module Proportion of patients with AMI receiving educational pamphlet
Penetration	Proportion of ED triage nurses using red triage cards Proportion of ED physicians using pocket cards Proportion of ED physicians successfully completing training module Proportion of AMI patients reading educational pamphlet
Costs	Total costs of intervention, including costs of printing educational pamphlets, pocket cards, red triage cards, and congratulatory certificates over the course of the pilot trial

^a^Primary pilot trial outcome.

^b^AIM: acceptability of intervention measure.

^c^ED: emergency department.

#### Secondary Outcome: Feasibility

The feasibility of MIMC will be assessed through several methods. First, the 4-questions FIM instrument [29], integrated into surveys administered to KCMC ED providers, will assess feasibility. The FIM instrument measures feasibility as an implementation outcome of an intervention, and like the AIM instrument, has exhibited substantial validity [29]. Administered in survey format, responses to FIM questions will be measured using a 5-point Likert Scale and will be scored in the same manner as the AIM instrument described above.

Feasibility will further be evaluated from the qualitative data obtained from in-depth interviews with key stakeholders. Additional quantitative measures of feasibility will be obtained from observational data collected by the research team, including the total number of red “ACS Suspect” triage cards needing replacement due to loss or damage, the number of pocket cards requiring replacement, and the amount of time ED physicians and nurses took to complete the web-based training module (as self-reported on the provider survey).

#### Secondary Outcome: Fidelity

Implementation fidelity is defined by the extent to which an intervention is delivered as intended [44]. The fidelity of MIMIC will be assessed based on compliance with the intervention’s 5 components. To gauge fidelity of the triage card component, the proportion of patients with chest pain or shortness of breath who are flagged with red triage cards will be observed (with the denominator being the total number of ED patients with chest pain or shortness of breath enrolled during the trial period). Similarly, measures related to pocket cards and the training module will be determined by the proportion of ED physicians who receive a pocket card and bring it to work at least once, and the number of ED staff who begin the web-based training module, respectively. These proportions will be determined by direct observation. Finally, the proportion of patients with a documented diagnosis of AMI receiving an educational pamphlet will be used as a measure of fidelity to MIMIC’s patient education component.

#### Secondary Outcome: Penetration

Penetration will be measured by the proportion of ED triage nurses who use the red triage cards, the proportion of ED physicians who bring their pocket cards to work each day, the proportion of ED providers who complete the training module, and the proportion of patients with AMI who report having read their educational pamphlet by the time of their 30-day follow-up.

#### Secondary Outcome: Costs

All costs needed to implement MIMIC will also be monitored as a secondary implementation outcome. Expected costs will include printing educational pamphlets, pocket cards, triage cards, and congratulatory certificates. All amounts will be converted from Tanzanian shillings to USD for analysis.

### Efficacy Outcomes

#### Secondary Outcome: AMI Testing in the ED

Early screening with ECGs and troponin testing has been shown to reduce morbidity and mortality among patients with AMI [45,46]. Therefore, the proportion of ED patients with chest pain or shortness of breath who receive an ECG and troponin testing in the ED will be evaluated as one efficacy measure of MIMIC. This proportion will be compared with the proportion of ED patients undergoing ECG and troponin testing for the period prior to implementation of the MIMIC intervention.

#### Secondary Outcome: Aspirin Treatment

Another efficacy measure will be the proportion of patients with AMI who are treated with aspirin in the ED. This proportion will be compared with preintervention observational data, as described above. For study purposes, patients with AMI will be defined as any patient with a physician-documented diagnosis of AMI or any patient meeting objective ECG and troponin criteria for AMI, as per the Fourth Universal Definition of Myocardial Infarction Guidelines [47]. Although not a formal outcome of this study, we will also observe the proportion of patients with AMI who receive heparin, P2Y12 inhibitors, and thrombolytics to inform future studies of the MIMIC intervention.

#### Secondary Outcome: 30-Day Mortality

Thirty-day mortality for patients with a documented diagnosis of AMI will be calculated and compared with preintervention observational data. Participants lost to follow-up will be excluded from this analysis.

#### Secondary Outcome: 30-Day Aspirin Use

The proportion of surviving participants with documented AMI diagnosis who report ongoing daily aspirin use at 30-day follow-up will also be a secondary efficacy outcome. This proportion will be compared with preintervention observational data; participants who died or were lost to follow-up will be excluded from this analysis.

### Statistical Analysis

The main objective of this trial is to assess the acceptability of MIMIC, which will determined by calculating the mean AIM score. AIM scores will be gathered from all participants, including patients, ED providers, and administrators, and then divided by the total number of trial participants. Importantly, the AIM scores from participants will be assumed to be normally distributed and independent of each other. The overall AIM score will fall within a range of 1 to 5, with higher scores indicating greater acceptability. An overall mean AIM score of 4.0 will be considered to indicate acceptability, as described above.

The acceptability of MIMIC will also be examined via qualitative analysis of the content of in-depth interviews across stakeholders. Two independent coders will conduct thematic analyses of interview transcripts using NVivo (version 14; Lumivero) in an iterative cycle of coding, discussion, and consensus-building. The coders will be assumed to be consistent and reliable in their coding process, and the findings from the qualitative analysis will be assumed to be accurate representations of participants’ views. Coders will use an inductive analytic approach, and disagreements among coders will be resolved by discussion and consensus-building. In the event a coding disagreement cannot be resolved by discussion, a third member of the coding team will serve as the tiebreaker. These thematic analyses will be guided by the Theoretical Framework for Acceptability [30].

Other implementation outcomes, such as fidelity and penetration, will be characterized by the proportion of participants receiving or complying with an intervention out of the total number of participants, as described above. In calculating these proportions, it will be assumed that the observations are independent of one another. Feasibility will be assessed through the FIM score mean and SD. To assess feasibility and costs implementation outcomes, the total number of resources allocated will be determined in place of proportions, as described above.

Efficacy outcomes will be used to assess the preliminary effectiveness of MIMIC. Pre-post analyses will be used to compare performance metrics from before and after MIMIC rollout to estimate the effect of the intervention on the 4 secondary efficacy outcomes listed in Table 2. Performance metrics from the preintervention and postintervention periods will be assumed to be independent of each other, and the conditions under which data were collected from each period will be assumed to be comparable.

To estimate effect sizes of the intervention, proportions of patients achieving each of the 4 key target efficacy outcomes will be compared from the preintervention period and postintervention period via Pearson chi-square test [48]. Preliminary effectiveness will be reported as odds ratios with corresponding 95% CIs constructed directly from two-by-two contingency tables. Preintervention data will be taken from an observational study conducted immediately prior to MIMIC rollout (February 1, 2023 to September 1, 2023). No a priori assumptions will be made regarding effect size of MIMIC on any of the 4 outcomes.

**Table 2:** Efficacy outcomes and measures.

Efficacy outcomes	Measures
AMI^a^ diagnostic testing	Proportion of patients with chest pain or dyspnea receiving ECG^b^ and troponin testing in the ED^c^
AMI Care in ED	Proportion of patients with AMI treated with aspirin in the ED
Thirty-day mortality	Proportion of patients with documented diagnosis of AMI who die within 30 days of presentation
Outpatient aspirin use at follow-up	Proportion of patients with AMI taking daily aspirin 30 days after initial presentation

^a^AIM: acceptability of intervention measure.

^b^ECG: electrocardiogram.

^c^ED: emergency department.

### Sample Size

No a priori assumptions will be made about the acceptability of the MIMIC intervention. There are currently approximately 70 total physicians and nurses working in the KCMC ED, and we will aim to administer the provider survey to at least 90% of the staff (63 total participants). Recent studies of the acceptability of other health care quality improvement interventions using the AIM instrument have reported SDs ranging from 0.4 to 0.8 when reporting the AIM score on a 5-point scale [49-51]. Assuming an SD of 0.6 in our study, a sample size of 63 participants would allow us to estimate the mean AIM score with a margin of error of ±0.2 with 95% CI.

Our observational study of ED care during the MIMIC pilot trial will be conducted for 1 year. Based on prior observational studies in the KCMD ED [11,12], we expect to enroll 400 patients with chest pain or dyspnea during the trial. The data generated from this pilot study will inform sample size calculations for a future clinical trial.

For qualitative interviews, approximately 35 participants will be recruited. This cohort will include 10 patients with AMI, 10 physicians, 10 nurses, and 5 hospital administrators. Final sample size will be determined by thematic saturation.

### Data Management and Safety Monitoring

Data from electronic health records and clinical care observations will be systematically collected on encrypted tablets using the Open Data Kit platform (version 2024.1.3; ODK) and uploaded directly to a secure, encrypted server at KCMC. Interview transcripts will be recorded and stored on encrypted digital devices accessible exclusively to authorized research personnel. Thematic analysis of these transcripts will be conducted using the NVivo software, with measures in place to encrypt and safeguard data. All data will be password-protected to maintain strict confidentiality. Prior to accessing the data within these systems, research personnel will undergo prerequisite training to ensure compliance with data security protocols. The Principal Investigator (JTH) will oversee data management by conducting weekly queries and reviewing inputted data for accuracy and completeness.

Following MIMIC rollout, stringent safety monitoring measures will be put in place to ensure the safety of the intervention. Importantly, MIMIC will be implemented by ED providers rather than by research staff, so the primary focus on safety monitoring will be to ensure that research activities do not disrupt the regular operations of the ED. Therefore, research personnel will undergo mandatory training to strictly observe and refrain from interfering with the clinical care provided to patients with AMI within the KCMC ED. Furthermore, all interviews will be scheduled during break periods, mealtimes, or at the conclusion of shifts to minimize any potential disruptions to patient care.

The principal investigator (JTH) and local coinvestigator (FMS) will be responsible for conducting safety reviews of the MIMIC intervention and addressing any threats to patient safety that may arise. Any instances of serious adverse events occurring during the implementation of MIMIC will be documented and reported to the local institutional review board in accordance with established safety and ethics protocols.

A formal interim analysis will be conducted 6 months into the pilot trial. At this point, the investigator team (JTH, GSB, NMT, FMS, JPB, and BTM) will analyze intervention fidelity data and patterns of care for four key care processes: (1) the proportion of patients presenting with chest pain or shortness of breath to the ED who undergo testing for AMI, (2) the proportion of patients with AMI who receive aspirin in the ED, (3) the proportion of patients with AMI who are alive at 30 days, and (4) the proportion of patients with AMI who are taking aspirin at 30 days. As this study involves a quality improvement intervention, we do not anticipate any adverse events. If any adverse events do occur, they will be reported, as required, to the Institutional Review Boards at Duke Health, KCMC, Tanzania National Institute for Medical Research, and the National Institutes of Health. We do not anticipate any changes to the trial protocol, but if these occur, these changes will be reported to the Institutional Review Boards at Duke Health, KCMC, Tanzania National Institute for Medical Research; the ClinicalTrials.gov record will also be updated.

### Ethical Considerations

Ethical approval for this study was obtained from the Tanzania National Institute for Medical Research (NIMR/HQ/R.8a/Vol. IX/2436, Version 7, February 23, 2021), KCMC (Proposal 893, Version 7, December 21, 2020), and Duke Health (Pro00090902, Version 1.7, September 16, 2020). All study procedures will be in accordance with the Helsinki Declaration of 1975, as revised in 2000. Research assistants will obtain written, informed consent from all research participants, including patients and ED providers, prior to their enrollment. Consent forms will be available in both English and Swahili to ensure they are understood by participants. Participation will be entirely voluntary, and patients all patients will reserve the right to opt out of the study at any time. Participant privacy will be respected, and no identifying information about participants will be shared publicly. Research personnel will observe MIMIC interventions and will refrain from disrupting clinical care. ED staff who participate in in-depth interviews and staff surveys will be compensated 5000 Tanzanian shillings (approximately US $2) in exchange for their time completing interviews and surveys. All data will be anonymized and stored in a secure, encrypted system accessible only to authorized research personnel. All clinically actionable findings identified via patient health records and observations of patient care will be reported to the primary clinical team at KCMC. In addition, all serious adverse events occurring during the implementation of MIMIC will be reported to the local institutional review board.

### Dissemination

Findings of this pilot trial will be disseminated among KCMC ED providers and hospital administrators via in-person conference meetings and presentations. Findings will also be published in peer-reviewed journals to inform broader audiences of implementation science interventions to improve AMI care in resource-limited settings. Trial results will be shared with local and national representatives of the Ministry of Health in Tanzania. If MIMIC is found to be acceptable, the findings from this study will inform a stepped-wedge, cluster-randomized trial to assess the effectiveness and scalability of the intervention across multiple health care facilities in Tanzania.

## Results

This study was funded in February 2021 and received its final ethical approval from the Tanzania National Institute for Medical Research in February 2021. The KCMC ED staff began implementing the MIMIC intervention on September 1, 2023. As of April 2024, a total of 275 adult patients with chest pain or shortness of breath have been enrolled in the study. Enrollment is expected to be completed by September 2024 and all analyses are planned to be completed by October 2024. The first results are expected to be published by December 2024.

## Discussion

With respect to implementation outcomes, we anticipate MIMIC to demonstrate high levels of acceptability, feasibility, and fidelity in the current trial. As for efficacy outcomes, we anticipate that MIMIC will enhance diagnostic testing rates among potential patients with AMI, increase the proportion of patients receiving aspirin, reduce 30-day mortality rates, and boost the proportion of patients with AMI adhering to aspirin 30 days post enrollment. More broadly, we anticipate that this will be the first study to investigate an evidence-based intervention aimed at improving AMI care in SSA. The study will also be the first in the region to assess an intervention aimed at increasing aspirin administration to patients with AMI—a World Health Organization “Best Buy” for mitigating noncommunicable disease morbidity and mortality [9].

However, it will be necessary to interpret the trial’s findings in light of its limitations. These include (1) the lack of a control arm, which limits the rigor of efficacy analyses, (2) use of pre-post comparisons for effectiveness outcomes, which may be subject to time-related confounders, and (3) unknown generalizability to settings beyond Tanzania.

If MIMIC demonstrates acceptability and effectiveness, then it may catalyze further research to implement similar interventions for emergency AMI care at a broader, more scalable level. Furthermore, the findings from this study will demonstrate the potential for rigorous implementation of scientific methods to enhance AMI care in SSA, especially with regard to aspirin administration. More broadly, this study will add to the growing body of research focusing on the effective and feasible translation of evidence-based procedures into practice within resource-limited settings.

### Conclusions

In light of recent evidence suggesting that uptake of evidence-based AMI care is lacking in Tanzania, MIMIC holds promise as a valuable intervention that can improve AMI care delivery in the country. If MIMIC demonstrates successful implementation and efficacy outcomes in this pilot trial, then the results could pave the way for broader implementation of MIMIC in Tanzania and elsewhere in SSA, helping to mitigate the growing burden of AMI in the region.

## Appendix

Multimedia Appendix 1 MIMIC pilot trial provider survey. MIMIC: multicomponent intervention to improve acute myocardial infarction care.

Multimedia Appendix 2 Peer-review reports by the Mentored Patient-Oriented Research Review (MPOR) Committee - Heart, Lung, and Blood Initial Review Group - National Heart, Lung, and Blood Institute (NHLBI) (National Institutes of Health, USA).

## Abbreviations

AIM: acceptability of intervention measure
AMI: acute myocardial infarction
ECG: electrocardiogram
ED: emergency department
FIM: feasibility of intervention measure
KCMC: Kilimanjaro Christian Medical Centre
MIMIC: multicomponent intervention to improve acute myocardial infarction care
SSA: sub-Saharan Africa

## Data Availability

Data collected over the course of the pilot trial on MIMIC will be publicly available upon reasonable request to the corresponding author.
