# Supplementary material for: Multicomponent Intervention to Improve Acute Myocardial Infarction Care in Tanzania: Protocol for a Pilot Implementation Trial
Source: JMIR Res Protoc. 2024 Sep 24;13:e59917. doi: 10.2196/59917 (PMC11462132; doi:10.2196/59917)
Supplement: Multimedia Appendix 1 [file resprot_v13i1e59917_app1.docx]

MIMIC Pilot Trial Provider Survey

Introduction: As you know, the KCMC EMD Team has worked to create a quality improvement intervention to improve MI care, called “MIMIC”. This intervention consisted of the following: (1) use of special red “Emergency ACS” cards by the triage nurses to indicate patients with possible ACS, (2) an online module with ACS refresher training for all EMD providers, (3) pocketcards for EMD doctors to use to help them remember ACS diagnosis and treatment, (4) educational pamphlets for patients with ACS, and (5) a designated physician champion and nurse champion to help encourage the team to improve ACS care.

1. Today’s date/**Tarehe ya leo:** ______
2. Surveyor initials/**Ufupisho wa majina ya mchunguzi:**____________
3. Respondent provider type/**Daraja/Elimu ya mtoa huduma anayejibu:**

□ Specialist MD, specify/ **Dactari bingwa, Taja/ainisha**: ____

□ General Practitioner MD/**Daktari wa kawaida**

□ CO

□ RN

□ Intern/**Intern**

Acceptability of Intervention Measure (AIM)

1. Overall, the MIMIC intervention to improve ACS care meets my approval [Kwa jumla, nakubali ule mradi wa “MIMIC” kuboresha matibabu ya shambulio la moyo]

□ Strongly Agree/**Nakubali kabisa** □ Agree/**Nakubali**  □ Neutral/don’t know/**Sijui** □ Disagree/**Sikubali**  □ Strongly Disagree/ **Sikubali kabisa.**

1. Overall, the MIMIC intervention to improve ACS is appealing to me [Kwa jumla, ule mradi wa “MIMIC” kuboresha matibabu ya shambulio la moyo unanivutia]

□ Strongly Agree/**Nakubali kabisa** □ Agree/**Nakubali**  □ Neutral/don’t know/**Sijui** □ Disagree/**Sikubali**  □ Strongly Disagree/ **Sikubali kabisa.**

1. Overall, I like the MIMIC intervention to improve ACS [Kwa jumla, nimependa ule mradi wa “MIMIC” kuboresha matibabu ya shambulio la moyo]

□ Strongly Agree/**Nakubali kabisa** □ Agree/**Nakubali**  □ Neutral/don’t know/**Sijui** □ Disagree/**Sikubali**  □ Strongly Disagree/ **Sikubali kabisa.**

1. Overall, I welcome the MIMIC intervention to improve ACS [Kwa jumla, nimefurahi kwamba ule mradi wa “MIMIC” kuboresha matibabu ya shambulio la moyo umekuwepo]

□ Strongly Agree/**Nakubali kabisa** □ Agree/**Nakubali**  □ Neutral/don’t know/**Sijui** □ Disagree/**Sikubali**  □ Strongly Disagree/ **Sikubali kabisa.**

Feasibility of Intervention Measure (FIM)

1. Overall, the MIMIC intervention to improve ACS care seems implementable.

□ Strongly Agree/**Nakubali kabisa** □ Agree/**Nakubali**  □ Neutral/don’t know/**Sijui** □ Disagree/**Sikubali**  □ Strongly Disagree/ **Sikubali kabisa.**

1. Overall, the MIMIC intervention to improve ACS care seems possible.

□ Strongly Agree/**Nakubali kabisa** □ Agree/**Nakubali**  □ Neutral/don’t know/**Sijui** □ Disagree/**Sikubali**  □ Strongly Disagree/ **Sikubali kabisa.**

1. Overall, the MIMIC intervention to improve ACS care seems doable.

□ Strongly Agree/**Nakubali kabisa** □ Agree/**Nakubali**  □ Neutral/don’t know/**Sijui** □ Disagree/**Sikubali**  □ Strongly Disagree/ **Sikubali kabisa.**

1. Overall, the MIMIC intervention to improve ACS care seems easy to use.

□ Strongly Agree/**Nakubali kabisa** □ Agree/**Nakubali**  □ Neutral/don’t know/**Sijui** □ Disagree/**Sikubali**  □ Strongly Disagree/ **Sikubali kabisa.**

Pocket cards

1. Did you receive an ACS pocket card?

□ Yes □ No

1. Did you use the ACS pocket card?

□ Yes □ No

1. If you didn’t use the ACS pocket card, why not?

□ I forgot □ I didn’t need it □ I didn’t think it was helpful □ it wouldn’t fit in my clothes

□ I didn’t like it □ other (specify): ___________

1. Did you lose your replacement card

□ Yes □ No

1. If yes, did you get a replacement card?

□ Yes □ No

1. Overall, I liked the ACS pocketcards

□ Strongly Agree/**Nakubali kabisa** □ Agree/**Nakubali**  □ Neutral/don’t know/**Sijui** □ Disagree/**Sikubali**  □ Strongly Disagree/ **Sikubali kabisa.**

Training module

1. Were you told to complete the online ACS training module?

□ Yes □ No

1. Did complete the ACS training module?

□ Yes □ No

1. If you didn’t complete the ACS training module, why not?

□ I forgot □ I didn’t think it would be helpful □ I had technical/internet issues

□ It was too difficult □ I didn’t have time □ I wasn’t told about it □ other (specify): ___________

19. How many minutes did you spend completing the online ACS training module? ____

1. Overall, I liked the online ACS training module

□ Strongly Agree/**Nakubali kabisa** □ Agree/**Nakubali**  □ Neutral/don’t know/**Sijui** □ Disagree/**Sikubali**  □ Strongly Disagree/ **Sikubali kabisa.**

**For nurses who work in Triage only:**

1. Did you use the red “Emergency ACS” triage cards?

□ Yes □ No

1. If not, why not?

□ I forgot □ I didn’t think it would be helpful □ I couldn’t find them/ran out

□ It was too difficult □ I didn’t have time □ I wasn’t told about it

□ I don’t know the symptoms of ACS □ other (specify): ___________

**For all providers:**

1. Overall, I liked the system of using the red “Emergency ACS” triage cards

□ Strongly Agree/**Nakubali kabisa** □ Agree/**Nakubali**  □ Neutral/don’t know/**Sijui** □ Disagree/**Sikubali**  □ Strongly Disagree/ **Sikubali kabisa.**

1. Overall, I liked having dedicated doctor and nurse champions for ACS quality improvement

□ Strongly Agree/**Nakubali kabisa** □ Agree/**Nakubali**  □ Neutral/don’t know/**Sijui** □ Disagree/**Sikubali**  □ Strongly Disagree/ **Sikubali kabisa.**

1. Overall, I liked the educational pamphlets for the patients

□ Strongly Agree/**Nakubali kabisa** □ Agree/**Nakubali**  □ Neutral/don’t know/**Sijui** □ Disagree/**Sikubali**  □ Strongly Disagree/ **Sikubali kabisa.**

1. **Do you have any other comments? __**
