# Supplementary material for: Multicomponent Intervention to Improve Acute Myocardial Infarction Care in Tanzania: Protocol for a Pilot Implementation Trial
Source: JMIR Res Protoc. 2024 Sep 24;13:e59917. doi: 10.2196/59917 (PMC11462132; doi:10.2196/59917)
Supplement: Multimedia Appendix 2 [file resprot_v13i1e59917_app2.pdf]

SUMMARY STATEMENT
(Privileged Communication)

PROGRAM CONTACT:
Nicole Redmond MD
301-435-0379
nicole.redmond@nih.gov

Release Date: 09/10/2020
Revised Date:

Application Number: 1 K23 HL155500-01

Principal Investigator

HERTZ, JULIAN T

Applicant Organization: DUKE UNIVERSITY

Review Group: MPOR (OA)
NHLBI Mentored Patient-Oriented Research Review Committee

Meeting Date: 07/23/2020
Council: OCT 2020
Requested Start: 09/01/2020
RFA/PA: PA19-118
PCC: HHCP N

Project Title: Developing an intervention to improve quality of myocardial infarction care in northern Tanzania
SRG Action: Impact Score:18
Next Steps: Visit https://grants.nih.gov/grants/next_steps.htm
Human Subjects: 30-Human subjects involved - Certified, no SRG concerns
Animal Subjects: 10-No live vertebrate animals involved for competing appl.
Gender: 1A-Both genders, scientifically acceptable
Minority: 5A-Only foreign subjects, scientifically acceptable
Age: 3A-No children included, scientifically acceptable

Project Year	Direct Costs Requested	Estimated Total Cost
1	154,650	167,022
2	154,830	167,216
3	154,500	166,860
4	154,500	166,860
5	154,500	166,860
TOTAL	772,980	834,818

ADMINISTRATIVE BUDGET NOTE: The budget shown is the requested budget and has not been adjusted to reflect any recommendations made by reviewers. If an award is planned, the costs will be calculated by Institute grants management staff based on the recommendations outlined below in the COMMITTEE BUDGET RECOMMENDATIONS section.

1 K23 HL 155500 - 01 HERTZ, JULIAN

RESUME AND SUMMARY OF DISCUSSION:
In this new K23 application, Dr. Julian Hertz, an emergency medicine physician, proposes a research career development plan with a training focus on implementation science, intervention development, and clinical trial design toward improving MI care of patients in low-resource settings – Tanzania in this proposal. The Candidate is exceptional. He has demonstrated a commitment to this line of research/care throughout his career. He's a two-time Fogarty fellow. He has a MS in Global Health. The Mentors are outstanding and supportive of the Candidate. The Career Development Plan would have been strengthened with benchmarks of productivity. Duke University and the collaborators in Tanzania provide an outstanding research infrastructure. The Research plan builds on strong preliminary data. It is innovative in including provider perspective in its qualitative aim. Overall, there was very high enthusiasm for this new application from an outstanding, focused, well-trained young investigator.

DESCRIPTION (provided by applicant):
Myocardial infarction (MI) is a leading cause of death worldwide. There is a critical need to improve uptake of evidence-based care for MI in resource-limited settings. This proposal presents a research career development program to 1) identify barriers to evidence-based MI care in Tanzania, 2) develop a multicomponent intervention to improve care by adapting proven strategies from other settings, and 3) assess the feasibility, acceptability, and potential effectiveness of the intervention. The candidate, an Assistant Professor of Surgery at Duke University and emergency medicine physician, has experience conducting research regarding MI in Tanzania. The high MI-associated mortality he identified as an NIH Fogarty Global Health Fellow led him to seek additional training in implementation science, intervention development, and clinical trial design. Working with a diverse mentoring committee of experts in implementation research, clinical trials, and global cardiovascular disease, as well as Tanzanian colleagues with whom he has collaborated for the past ten years, he will build upon his prior study of MI in northern Tanzania. The proposed activities will equip the candidate with a combination of skills in global emergency implementation research and position him to become a successful independent investigator operating at the intersection of global health, emergency medicine, and implementation science. Growing evidence suggests that in sub-Saharan Africa (SSA), MI is common, frequently misdiagnosed, rarely treated with evidence-based therapies, and associated with high mortality. Preliminary research conducted by the candidate in Tanzania found that 22% of patients presenting to the emergency department (ED) with typical symptoms have an acute MI, but 90% of acute MI cases are missed by ED physicians. The candidate's research found a 30-day mortality rate following acute MI of 43%--more than six-fold higher than the mortality rate in the United States. Such poor MI outcomes are likely related to low uptake of evidence-based MI care; for example, the candidate's preliminary data found that only 23% of patients with acute MI were treated with aspirin—a WHO "best buy" for reducing noncommunicable disease mortality. Despite these alarming data, the barriers to evidence-based MI care in SSA remain largely unexplored and locally-tailored interventions to improve MI care in resource limited settings are sorely lacking. There is an urgent need to identify barriers to MI care and to develop locally-appropriate interventions that will improve MI outcomes in low-income settings like Tanzania. This proposal will bridge this knowledge gap by using an approach rooted in implementation science. Specifically, this study will 1) identify barriers to evidence-based diagnosis and treatment of MI in northern Tanzania, 2) develop a multicomponent tailored intervention to improve MI care, and 3) determine feasibility, acceptability, and potential effect of a tailored intervention on MI care. This study will be an important first step towards reducing excess MI-associated morbidity and mortality in resource-limited settings.

PUBLIC HEALTH RELEVANCE:
Evidence suggests that in sub-Saharan Africa myocardial infarction (MI) is associated with very high mortality, but locally-appropriate interventions to improve uptake of evidence-based care are lacking. This study will identify barriers to MI care, develop a tailored multicomponent intervention to improve care, and assess the feasibility, acceptability, and preliminary effectiveness of this tailored intervention. The

results from this study will lead directly to clinical trials to test interventions to reduce mortality from myocardial infarction in resource-limited domestic and global settings.

CRITIQUE 1:

Candidate: 1
Career Development Plan/Career Goals /Plan to Provide Mentoring: 3
Research Plan: 4
Mentor(s), Co-Mentor(s), Consultant(s), Collaborator(s): 1
Environment Commitment to the Candidate: 1

Overall Impact:
This is a first-time application for an Emergency Medicine physician-scientist with exceptional training in global health with long-term goal to improve emergency care in resource poor settings. The candidate has been very productive with research publications. The mentorship team is exceptional and has a great track record of working with the candidate and together. The career development is very strong. The research proposed has the potential to substantially improve the diagnosis and treatment of myocardial infarction (MI) in Tanzania. There are a few weaknesses that dampened overall enthusiasm. The candidate does not adequately address time spent in U.S. vs. Tanzania and how research operations will occur from a distance. There are major innovative components of the research plan. Finally, there is little description of how provider and patient characteristics will be captured and incorporated into analyses.

1. Candidate:
Strengths
- The candidate has substantial expertise in global health with practical experience in Haiti and Tanzania (Fogarty International Clinical Research Scholarship, a fellowship in Global Emergency Medicine.
- The candidate has a Master's Degree in Global Health.
- The candidate completed the NIH Fogarty Global Health Fellowship.
- The candidate holds a Duke Center for AIDS Research grant.
- The candidate has extensive partnerships and collaborators in Tanzania.
- The candidate has over 30 peer-reviewed publications, with greater than 17 as first-author.

Weaknesses
- None

2. Career Development Plan/Career Goals & Objectives:
Strengths
- The candidate's career goal is to use implementation science research to improve the quality of emergency care in resource-limited settings.
- The candidate will use this award to receive training not previously gained. The major areas of focus will be implementation science, intervention development, and trial design.
- The training activities proposed are aligned with the candidate's career goals and demonstrate diversity in the types of experiences – intensive courses, online courses, in-person courses.
- The frequency of meetings with mentors is appropriate.

Weaknesses

- No benchmarks for productivity (i.e., number of manuscripts to be submitted throughout the award) are provided.
- There is some contradiction in when the R01 will be submitted. It says year 5 once but year 4 everywhere else.
- The candidate does not address travel and research oversight during the award, i.e., how much time will the candidate spend in Tanzania, who will be in charge when the candidate is not there, how will communication occur with a distant research team.

3. Research Plan:
Strengths
- The specific aims are well articulated and logically build upon each other.
- The scientific premise for the proposed work is well stated. There are few studies assessing barriers to diagnosis and treatment of MI in SSA. Evidence-based interventions are needed to improve diagnosis and treatment.
- The candidate presents preliminary data that informs the current proposal.
- A mixed methods approach will be used to develop an intervention.
- The proposed work will be guided by the Consolidated Framework for Implementation Research (CFIR).

Weaknesses
- The proposed work does not have any major innovative pieces.
- There is little detail on capturing characteristics of providers and patients and how those variables will be addressed in any analyses.

4. Mentor(s), Co-Mentor(s), Consultant(s), Collaborator(s):
Strengths
- The candidate has published with the mentors.
- The mentors have complementary experience in international trials, implementation science, and myocardial infarction.

Weaknesses
- None

5. Environment and Institutional Commitment to the Candidate:
Strengths
- Duke has exceptional resources as a research university.
- The candidate has a strong network of collaborators and research staff in Tanzania.
- The institutional letter is very strong.

Weaknesses
- None

CRITIQUE 2:

Candidate: 1
Career Development Plan/Career Goals /Plan to Provide Mentoring: 2
Research Plan: 3
Mentor(s), Co-Mentor(s), Consultant(s), Collaborator(s): 1

Environment Commitment to the Candidate: 1

Overall Impact:
This application is from a very impressive and productive physician scientist who demonstrates a palpable commitment to the proposed area of research. The applicant has an impressive record of history in-country collaborating on projects at the proposed research site. The mentorship team is excellent as is the institutional environment and the research infrastructure through the KCMC-Duke collaboration is stellar and demonstrates the feasibility of the partnership. There is a strong set of preliminary data. The research methods are appropriate to the proposed research aims. There are a few weaknesses in the candidate plan and research plan, which are detailed below.

1. Candidate:
Strengths
- Faculty Clinician of the Year Award, Duke Emergency Medicine (2018)
- Faculty Teacher of the Year Award, Duke Emergency Medicine (2017)
- Fogarty twice.
- 30 pubs with mentors, 17 are first authored.
- Interest in global CV research.
- Focus on underserved pops.
- Candidate has been conducting research at KCMC for 10 years. 30 pubs stemming from research in Tanzania, and has a long history of conducting global health research in-country.
- During med school at duke spent 2 years in Tanzania conducting clinical studies with the Duke-KCMC collaboration via his Fogarty clinical research scholarship.
- EM doc wanting career in IS in resource limited settings to improve emergency care. Commitment to area of work is clearly demonstrated.
- During Fogarty fellowship got experience with clinical research skills, data analysis, and ms writing.
- Master’s at Duke: study design quant and qual research; then back to Tanzania for a year conducting clinical research focused on diagnosis of chronic disease in northern Tanzania including MI.
- Has spent a lot of time in-country and in low-resourced settings more broadly. Strong research background. Commitment to improving emergency care.
- Assist Prof now at Duke in EM. Also, faculty appointment at DGHI. Access to free and unlimited stats support from research design and analytics core, and admin support. Been conducting research in Tanzania for 9 years. Strong collaborative relationships with key local stakeholders.
- Has ongoing studies in Tanzania: long-tern f/u on patients with acute MI in northern Tanzania; burden of MI among HIV-infected Tanzanian adults.

Weaknesses
- None noted.

2. Career Development Plan/Career Goals & Objectives:
Strengths
- Long-term goal is to improve MI outcomes in SSA.
- This application: improve MI care in northern Tanzania. Preliminary work identified potential targets for improvement: provider training, intentional consideration of diagnosis of MI (MI testing), local treatment protocols, aspirin.

- Made strong case for need for additional training: implementation research methods, intervention development, clinical trial design, mix of training activities: courses, seminars, conferences, mentorship, direct research.
- IS training: NIH training institute for dissemination and implementation research in health incudes mentorship and a training session at the NIH. Strong complement of IS courses spread out over years 1-4/5.
- Conferences
- Hands on research
- Great training in clinical research and intervention dev: Duke Translational Research Center of BSS interventions: longitudinal training experiential workshops 4x per year.
- Grant writing workshop in year 4.
- LEADER seminar series at Duke to provide training on managing research teams. Writing from reader's perspective workshop from leader in scientific writing (Gopen).

Weaknesses

- At what point do you plan to broaden beyond Tanzania since that is a goal? How will this award help you with that longer-term goal? This was mentioned as a career goal. What are the plans for this now or later?
- Is there overlap between Thielman (international clinical trials, particularly in Tanzania, and will provide invaluable mentorship regarding study design, conduct, and data analysis) and Bartlett? Professor of Medicine at the Kilimanjaro Christian Medical University. Extensive network of collaborations with key stakeholders in Tanzania will be an essential asset in the conduct and success of the proposed study. Will guide clinical trial design). Would like to see a clearer distinction between the two in terms of their mentorship of the trainee. What is the distinction between what they will each contribute to the training plan?

3. Research Plan:

Strengths

- Preliminary data shows high MI mortality in SSA (northern Tanzania) and lack of uptake of evidence-based interventions (of those diagnosed, low % being treated with evidence-based approaches such as aspirin administration) and a high rate of missed cases and establishes need to identify barriers to uptake and identify strategies appropriate for low resource settings. Also, high 30-day mortality rate.
- Preliminary qual data from providers identifies potential intervention targets: Inadequate provider training, failure to consider the diagnosis of MI, lack of local disease burden data, absent locally-relevant treatment protocols, and limited patient education were all cited by providers as important barriers to care.
- Prelim data only assessed provider perspectives. Need a more comprehensive study to assess full range of barriers to care and identify reasonable interventions. Mixed methods approach to identify barriers will improve understanding and likely be more comprehensive and allow for better quant descriptive data for informing intervention development. and will focus on various stakeholder groups not just providers as most prior studies have done. Well-rounded group of stakeholders: patient, providers, administrators, policy makers.
- Existing clinical research infrastructure at Kilimanjaro Christian Medical Center (KCMC) and surrounding healthcare facilities.
- Gap in many low resourced settings is not how to treat but how to implement. Evidence based care guidelines exist, but implementation is poor.

- Strong existing relationship with KCMC and other local stakeholders. PI helped develop MI diagnostic and treatment capacity at KCMC. Tertiary care center with all of northern Tanzania as catchment area. Preliminary data was conducted at KCMC so established relationship.
- Pre-post design to compare MI care data to estimate effectiveness and estimate effect sizes for subsequent R01.
- Research impact: among the first to describe barriers and produce a testable intervention to improve MI care and outcomes in Tanzania and other resource limited settings. First study to apply IS approach to improve MI care in SSA. Critical gap and high potential impact of this research to fill this gap.
- Scientific premise: Relatively recent and drastic rise in MI incidence coupled with poor data. Now 4th leading COD in Tanzania. Lack of MI testing suggesting MI may be underdiagnosed. Patients with symptoms not getting screened when go to ED and low rate of patients with symptoms even seeking care. All suggests a very high burden of MI in Tanzania and mostly undiagnosed.
- Prelim data: Big diff in positive diagnoses between physician discretion and routine screening. routing screening helps (2% vs. 22%). High 30-day MR... almost 50% among the highest worldwide.
- Improving diagnosis and treatment of MI in developing countries in a global health priority (WHO, WHF).
- Studies investigating interventions to improve MI care in SSA big research gap.
- IS promising approach for improving MI care in SSA given its effectiveness in other resource-depleted settings.
- Prelim data also shows implementation of treatment protocol (aspirin) increases diagnosis six-fold and treatment with aspirin seven-fold.
- Use of CFIR will ensure a systematic approach to data collection for Aim 1. Will improve contextual understanding and contextually relevant intervention. CFIR proven record of success in LMIC.
- Existing cohort of patients, providers and administrators will be recruited from broad range of health care facilities, range of orgs for recruiting policy makers.
- Serial data collection qual will inform KAP survey development. KAP survey will build from previous survey used in northern Tanzania.
- Survey will be tailored to assess barriers in the 4 target areas (eg, Mi testing, aspirin administration, long term aspirin therapy).
- Effect size for power calculation informed from prior research.
- Cultural insider-outsider team for qual analysis. Will help with code-checking as validation strategy.
- Grounded theory approach.
- Interdisciplinary design team to develop intervention: ED docs, cardiologists, IS, administrators, nurses, patients.
- Will adapt proven interventions from other settings to local context based on info collected in Aim 1.
- Standardization in outcome assessment (fourth universal def of MI guidelines).
- ECGs will be judged by 2 independent physicians. 3rd will be brought in when there is disagreement.
- Wide range of stakeholders and facilities will improve generalizability and effectiveness.

- Builds on previous work as Fogarty fellow in same location and on same topic: conducted a study on the under-diagnosis of noncommunicable diseases in northern Tanzania, including myocardial infarction (MI)…described above.

Weaknesses

- What happens if you don't reach theoretical saturation?
- Aim 1: How will the multivariable logistic regression be adjusted? and how will those data be used in Aims 2 and 3? Not clear to me what the predictors and outcomes are. ("multivariate logistic regression will be used to determine associations between participant characteristics, such as stakeholder type, and predictors of responses to key KAP items, such as willingness to participate in an intervention to improve MI care (implementation measure of adoption)."
- What if you find that older patients and those with less education are less likely to participate?
- Describe ERIC.
- Describe FRAME.
- What is general rate of agreement for ECGs in low resourced settings? "ECGs will be adjudicated by two independent physician judges trained in either cardiology or emergency medicine; in cases of disagreement, a third physician judge will serve as the tie-breaker."
- Preliminary data demonstrates successful follow up rate from prior studies 98%. What was the f/u length of prior studies?

4. Mentor(s), Co-Mentor(s), Consultant(s), Collaborator(s):

Strengths

- Strong mentorship team: global clinical research, CVD, IS.
- Mentorship team who have existing relationships with KCMC -- been doing research with KCMC for over 40 years.
- PI has co-authored 11 manuscripts with 2 researchers from KCMC.
- Will work with two research assistants that have 21 years of experience conducting clinical research with KCMC-Duke collaboration. They have experience enrolling MI patients, IC, admin questionnaires, conducting ID interviews, patient follow up in the community.
- Use of Tanzanian researchers for data collection.
- Mentor Bartlett describes network of local collaborators in Tanzania that will work with the PI to ensure the resources needed to accomplish the research objectives. Bartlett will oversee clinical trial design and mentor in area of global health research. Duke global health institute, Duke Africa Initiative, and prof of med at KCM university. Awards for teaching and mentorship; Prof of Med at KCMU. Will provide mentorship in clinical trial design, career dev and global health research.
- Bloomfield has been working with PI for past 6 years, they've published 4 papers together with 3 others in press. Bloomfield: global CV health in SSA, previous work with candidate in Tanzania. Will provide mentor on CVD and clinical research design. Says candidate assessed as top 2 mentees over past decade. Enormous potential, scholarly productivity.
- Mmbaga (local collaborator): letter from director of research at KC research institute, research director for KCMC duke research collaboration, director of research at KCM university. Been conducting clinical research in Tanzania for over a decade. Strong commitment to providing whatever candidate needs locally to be successful.
- Thielman: international clinical trials in Tanzania. Mentorship on study design conduct and data analysis? Strong history of NIH funding and mentorship. Will meet weekly to discuss study progress, career development, trial design, data analysis and grant prep.

- Bettger: HSR, track record of mentoring K awardees in IS. Mentor on NHLBI K12 focuses on IS. IS co-I on NIH awards. Will provide mentorship in IS. will meet weekly.

Weaknesses
- None noted.

5. Environment and Institutional Commitment to the Candidate:
Strengths
- Established Duke-KCMC research collaboration.
- Incredibly strong research infrastructure in terms of capability of the KCMC and project personnel.
- Sakita: head of KCMC ED. Fully committed to providing support needed. Will provide space and resources for candidate and candidate's research team.
- DGHI: global health institute which houses Duke Tanzania operations. Invested in building strong research infrastructure at KCMC. Free access to DGHI research design and analysis core (8 biostatisticians and epi). Capability in qual, quant and mixed methods.
- Letter of institutional commitment Duke: commitment of protected time, office space, admin staff; support not contingent on grant.

Weaknesses
- None noted.

Study Timeline:
Strengths
- None noted.

Weaknesses
- Clinical trials in year 5. Is this too late to be useful for completing research aims?

CRITIQUE 3:

Candidate: 1
Career Development Plan/Career Goals /Plan to Provide Mentoring: 1
Research Plan: 1
Mentor(s), Co-Mentor(s), Consultant(s), Collaborator(s): 1
Environment Commitment to the Candidate: 1

Overall Impact:
This is an outstanding first submission K23 application for Dr. Julian Hertz, an emergency medicine physician dedicated to a career in implementation research in low-resource settings and global health. With an outstanding training background already, Dr. Hertz demonstrated a commitment to POR as an undergraduate at Princeton University, where he spent a year volunteering in a clinic providing emergency care in rural Haiti. From there he has excelled, completing a Fogarty International Clinical Scholarship in Tanzania while in medical school (on Deans' Merit Scholarship) at Duke. Following his residency in Emergency Medicine at Vanderbilt, he returned to Duke for a fellowship in Global Emergency Medicine and obtained a master's degree in global health. He spent another year in Tanzania on the NIH Fogarty Global Health Fellowship (completed 2019). He has been exceedingly productive, with over 30 peer review, high-impact publications in his short career in the field of global emergency medicine, and has established strong collaborations with faculty in Tanzania (as well as learning fluent Swahili). He has already earned numerous grants, awards and accolades, for research and leadership as well as faculty clinician of the year. He had organized a very impressive group of career mentors with whom he has

already published with, who are clearly committed to his success, and describe him as “the best mentee every worked with” and “stands out as one of the very best in terms of his commitment to research, demonstrated track record of productivity, and rapid mastery of research skills. His mentors illustrate a thoughtful mentorship plan that not only highlights Dr. Hertz’ potential to develop into a successful independently funded investigator, but lays out plans for career development that includes appropriate coursework in implementation science and global health, coupled with professional development opportunities (ie Dukes LEADER program, Early Career Grant Writing Program” etc), and a clear path to independence and R01 funding. The research plan targeting barrier to diagnosis and treatment of MI in Tanzania followed by implementation of tailored evidence-based interventions will likely lead to changes in practice that are impactful. Together with the excellent environment at Duke, the demonstrated gap in implementation science education needed for career development that a mentored career award will address, Dr. Hertz is an ideal candidate for this K23. It was an absolute delight to review such a strong application for an individual who is already on his way to leadership in his field. There is a paucity of emergency medicine physician scientists and few who have expertise in implementation science; Dr. Hertz is an ideal candidate to benefit from this sort of opportunity.

1. Candidate:

Strengths

- Stellar background, education and consistent dedication to POR since undergraduate work.
- Emergency Medicine research leadership rare; implementation science and global health interests fit growing need.
- >30 peer reviewed publications in young career.
- Numerous awards, fellowships, and accolades attesting to his unique potential as emerging leader in the field.

Weaknesses

- None

2. Career Development Plan/Career Goals & Objectives:

Strengths

- Didactic training in implementation science a gap in knowledge to be filled by K23.
- Professional Development plan includes leadership workshops, manuscript and grant writing.
- Mentorship guidance appropriate.
- Well-organized plan, team science approach.

Weaknesses

- None

3. Research Plan:

Strengths

- Highly impactful: MI is leading cause of death globally and underappreciated in sub-Saharan Africa (SSA); urgent need.
- Implementation science around MI and emergency care in SSA as described will save lives.
- Innovative as first study to test implementation of treatment in low resource setting in SSA.
- Years of relationship building and preliminary studies support current plan.
- Will set path for R01 application.

Weaknesses

- None

4. Mentor(s), Co-Mentor(s), Consultant(s), Collaborator(s):
Strengths

- Stellar mentorship team with track record of high-impact publication, grant funding, and collaboration with candidate.
- Overwhelmingly supportive of the candidate's career development.
- One of best mentorship plans I've reviewed for a K23 candidate; experienced mentors clearly dedicated to and impressed by this candidate.
- All areas of expertise covered (global health, implementation science, emergency medicine, cardiovascular disease – both in US and abroad in Tanzania).

Weaknesses

- None

5. Environment and Institutional Commitment to the Candidate:
Strengths

- Duke is outstanding environment to foster career development.
- Recourses available as faculty of Duke Global Health Institute (including unlimited statistical support, grant support and assistance with developing training programs); DGHI houses the Duke Tanzania operations.
- Kilimanjaro Christian Medical Centre-Duke University Research Collaboration – ideal collaboration for proposed work.
- 75% time guaranteed protected time, with dedicated administrative staff – independent of K23.

Weaknesses

- None

Study Timeline:
Strengths

- Feasible

Weaknesses

- None noted.

THE FOLLOWING SECTIONS WERE PREPARED BY THE SCIENTIFIC REVIEW OFFICER TO SUMMARIZE THE OUTCOME OF DISCUSSIONS OF THE REVIEW COMMITTEE, OR REVIEWERS' WRITTEN CRITIQUES, ON THE FOLLOWING ISSUES:

PROTECTION OF HUMAN SUBJECTS (RESUME): ACCEPTABLE

INCLUSION OF WOMEN PLAN (RESUME): ACCEPTABLE

INCLUSION OF MINORITIES PLAN (RESUME): ACCEPTABLE

INCLUSION OF AGE ACROSS THE LIFESPAN (RESUME): ACCEPTABLE; no children.

TRAINING IN THE RESPONSIBLE CONDUCT OF RESEARCH: ACCEPTABLE

RESOURCE SHARING PLANS: NOT APPLICABLE (NO RELEVANT RESOURCES): ACCEPTABLE

AUTHENTICATION OF KEY BIOLOGICAL AND/OR CHEMICAL RESOURCES: ACCEPTABLE

COMMITTEE BUDGET RECOMMENDATIONS: RECOMMENDED AS REQUESTED

Footnotes for 1 K23 HL155500-01; PI Name: Hertz, Julian T

NIH has modified its policy regarding the receipt of resubmissions (amended applications).See Guide Notice NOT-OD-18-197 at https://grants.nih.gov/grants/guide/notice-files/NOT-OD-18-197.html. The impact/priority score is calculated after discussion of an application by averaging the overall scores (1-9) given by all voting reviewers on the committee and multiplying by 10. The criterion scores are submitted prior to the meeting by the individual reviewers assigned to an application, and are not discussed specifically at the review meeting or calculated into the overall impact score. Some applications also receive a percentile ranking. For details on the review process, see http://grants.nih.gov/grants/peer_review_process.htm#scoring.

MEETING ROSTER

NHLBI Mentored Patient-Oriented Research Review Committee
Heart, Lung, and Blood Initial Review Group
NATIONAL HEART, LUNG, AND BLOOD INSTITUTE
MPOR (OA)
07/23/2020 - 07/24/2020

Notice of NIH Policy to All Applicants: Meeting rosters are provided for information purposes only. Applicant investigators and institutional officials must not communicate directly with study section members about an application before or after the review. Failure to observe this policy will create a serious breach of integrity in the peer review process, and may lead to actions outlined in NOT-OD-14-073 at https://grants.nih.gov/grants/guide/notice-files/NOT-OD-14-073.html and NOT-OD-15-106 at https://grants.nih.gov/grants/guide/notice-files/NOT-OD-15-106.html, including removal of the application from immediate review.

CHAIRPERSON(S)

LEDERER, DAVID J., MD
MEDICAL DIRECTOR
REGENERON PHARMACEUTICAL, INC.
COLUMBIA UNIVERSITY IRVING MEDICAL CENTER
NEW YORK, NY 10032

MEMBERS

ALLEN, AMANI M., MPH, PHD
ASSOCIATE PROFESSOR
DEPARTMENT OF COMMUNITY HEALTH SCIENCES
DIVISION OF EPIDEMIOLOGY
SCHOOL OF PUBLIC HEALTH
UNIVERSITY OF CALIFORNIA, BERKELEY
BERKLEY, CA 94720

AMIN, RAOUF S., MD
PROFESSOR AND DIRECTOR
DIVISION OF PULMONARY MEDICINE
CINCINNATI CHILDREN'S HOSPITAL MEDICAL CENTER
CINCINNATI, OH 45229

ANDERSON, CHERYL ANN MARIE, PHD, MPH
PROFESSOR AND INTERIM CHAIR
DIVISION OF PREVENTIVE MEDICINE
DEPARTMENT OF FAMILY AND PREVENTIVE MEDICINE
UNIVERSITY OF CALIFORNIA, SAN DIEGO
SCHOOL OF MEDICINE
LA JOLLA, CA 92093

ARRUDA-OLSON, ADELAIDE MARIA MARTINS, MD *
PROFESSOR
DEPARTMENT OF INTERNAL MEDICINE
DIVISION OF CARDIOVASCULAR DISEASES
MAYO CLINIC COLLEGE OF MEDICINE
ROCHESTER, MN 55905

BADR, M. SAFWAN, MD
PROFESSOR AND CHAIRMAN
DEPARTMENT OF INTERNAL MEDICINE
DIV. OF PULMONARY, CRITICAL CARE/SLEEP MEDICINE
WAYNE STATE UNIVERSITY
DETROIT, MI 48201

CHOI, SUNG, MD, MS, BS *
ASSOCIATE PROFESSOR
DIVISION OF GENERAL PEDIATRICS
PEDIATRICS & COMMUNICABLE DISEASES
THE CENTER FOR HEALTH COMMMUNICATIONS RESEARCH
UNIVERSITY OF MICHIGAN AT ANN ARBOR
ANN ARBOR, MI 48109-5941

CHRISTIE, JASON D., MD
PROFESSOR
DIVISION OF PULMONARY AND CRITICAL CARE MEDICINE
CENTER FOR CLINICAL EPIDEMIOLOGY AND BIOSTATISTICS
SCHOOL OF MEDICINE
UNIVERSITY OF PENNSYLVANIA
PHILADELPHIA, PA 19104

COHEN, ROBYN T., MD *
DIRECTOR
PEDIATRIC PUMONARY
& ALLERGY CLNIC
BOSTON UNIVERSITY MEDICAL CENTER
NEWTON, MA 02458

CROTHERS, KRISTINA A., MD *
ASSOCIATE PROFESSOR
UNIVERSITY OF WASHINGTON SCHOOL OF MEDICINE
HARBORVIEW MEDICAL CENTER
PULMONARY, CRITICAL CARE AND SLEEP MEDICINE
SEATTLE, WA 98104

DAMARLA, MAHENDRA, BA, MD *
PROFESSOR
DEPARTMENT OF PULMONARY DISEASES
CRITICAL CARE MEDICINE
JOHNS HOPKINS UNIVERSITY
BALTIMORE, MD 21224-6821

EISEN, HOWARD J., MD
CHIEF
HEART AND VASCULAR INSTITUTE
MILTON S. HERSHEY MEDICAL CENTER
PENNSYLVANIA STATE UNIVERSITY
HERSHEY, PA 17033

EVERHART, ROBIN S, PHD
ASSOCIATE PROFESSOR
DEPARTMENT OF PSYCHOLOGY
VIRGINIA COMMONWEALTH UNIVERSITY
RICHMOND, VA 23284

FLOOD, VERONICA H., MD
PROFESSOR
DIVISION OF HEMATOLOGY/ONCOLOGY
BLOOD RESEARCH INSTITUTE
BLOOD CENTER OF WISCONSIN
MEDICAL COLLEGE OF WISCONSIN
MILWAUKEE, WI 53226

FOLTA, SARA, PHD *
ASSOCIATE PROFESSOR
FRIEDMAN SCHOOL OF NUTRITION
SCIENCE AND POLICY
TUFTS UNVIERSITY
BOSTON, MA 02111

JIMENEZ, JUAN M., BS, MS, PHD *
PROFESSOR
DEPARTMENT OF MECHANICAL
INDUSTRIAL ENGINEERING
COLLEGE OF ENGINEERING
AMHERST, MA 01003

LINDER, MARK W., PHD *
ASSOCIATE PROFESSOR
DEPARTMENT OF PATHOLOGY
AND LABORATORY MEDICINE
SCHOOL OF MEDICINE
UNIVERSITY OF LOUISVILLE
LOUISVILLE, KY 40202

MARINO, BRADLEY S., MD
PROFESSOR
DEPARTMENT OF PEDIATRICS AND MEDICAL SCIENCES
NORTHWESTERN UNIVERSITY FEINBERG
SCHOOL OF MEDICINE
ANN & ROBERT LURIE CHILDREN'S HOSPITAL OF CHICAGO
CHICAGO, IL 60611-2991

MAU, MARJORIE K. LEIMOMI MALA, MD
PROFESSOR
DEPARTMENT OF NATIVE HAWAIIN HEALTH
JOHN A. BURNS SCHOOL OF MEDICINE
UNIVERSITY OF HAWAII AT MANOA
HONOLULU, HI 96813

MIYAMOTO, SHELLEY D., MD
ASSOCIATE PROFESSOR
DEPARTMENT OF PEDIATRICS-CARDIOLOGY
CHILDREN'S HOSPITAL COLORADO
UNIVERSITY OF COLORADO SCHOOL OF MEDICINE
AURORA, CO 80045

MORRIS, ALISON, MD
PROFESSOR
DIVISION OF PULMONARY, ALLERGY
AND CRITICAL CARE MEDICINE
DEPARTMENT OF MEDICINE
UNIVERSITY OF PITTSBURGH AT PITTSBURGH
PITTSBURGH, PA 15213

MORRIS, CLAUDIA R., MD
PROFESSOR
DEPARTMENT OF PEDIATRICS
DIVISION OF EMERGENCY MEDICINE
EMORY UNIVERSITY SCHOOL OF MEDICINE
ATLANTA, GA 30322

NIETERT, PAUL J., PHD
PROFESSOR OF BIOSTATISTICS
DEPARTMENT OF PUBLIC HEALTH SCIENCES
MEDICAL UNIVERSITY OF SOUTH CAROLINA
CHARLESTON, SC 29425

NOTH, IMRE, MD
PROFESSOR OF MEDICINE
DEPARTMENT OF MEDICINE
DIVISION OF PULMONARY & CRITICAL CARE MEDICINE
UNIVERSITY OF VIRGINIA SCHOOL OF MEDICINE
CHARLOTTESVILLE, VA 22908

PIPINOS, IRAKLIS ILIAS, MD, PHD
PROFESSOR
DEPARTMENT OF SURGERY
UNIVERSITY OF NEBRASKA MEDICAL CENTER
OMAHA, NE 68198

POLLACK, ANNA Z., MPH, PHD, BA *
PROFESSOR
DEPARTMENHT OF GLOBAL & COMMUNITY HEALTH
COLLEGE OF HEALTH & HUMAN SERVICES
GEORGE MASON UNIVERSITY
FAIRFAX, VA 22030

RAPHAEL, JEAN L., MD, MPH
ASSOCIATE PROFESSOR OF PEDIATRICS
DEPARTMENT OF PEDIATRICS
BAYLOR COLLEGE OF MEDICINE
HOUSTON, TX 77030

SAN JOSE ESTEPAR, RAUL, PHD
ASSOCIATE PROFESSOR
DEPARTMENT OF RADIOLOGY
BRIGHAM AND WOMEN'S HOSPITAL
HARVARD MEDICAL SCHOOL
BOSTON, MA 02115

SINHA, SHANTANU, PHD *
PROFESSOR
DEPARTMENT OF RADIOLOGY
SCHOOL OF MEDICINE
UNIVERSITY OF CALIFORNIA - SAN DIEGO
SAN DIEGO, CA 92121

SNYDER, EDWARD L., MD, MA, BA *
PROFESSOR AND DIRECTOR
DEPARTMENT OF LABORATORY MEDICINE
AND BLOOD BANK
YALE-NEW HAVEN HOSPITAL
YALE UNIVERSITY
NEW HAVEN, CT 06504

TESTANI, JEFFREY M, MD *
ASSOCIATE PROFESSOR
SECTION OF CARDIOVASCULAR MEDICINE
YALE UNIVERSITY
NEW HAVEN, CT 06520

VAN DAM, ROBERT M., PHD *
ASSOCIATE PROFESSOR
DEPARTMENT OF PHARMACOLOGY
CRUMP INSTITUTE MOLECULAR IMAGING (CIMI)
UNIVERSITY OF CALIFORNIA, LOS ANGELES
GEFFEN SCHOOL OF MEDICINE
LOS ANGELES, CA 90095-1770

VEVES, ARISTIDIS, DSC, MA, MD, MS *
RONGXIANG XU, MD PROFESSOR OF SURGERY, HARVARD MEDICAL SCHOOL
DIRECTOR, RONGXIANG XU CENTER FOR REGENERATIVE THERAPEUTICS
RESEARCH DIRECTOR
JOSLIN-BETH ISRAEL DEACONESS FOOT CENTER
BOSTON, MA 02215

WANG, THOMAS J., MD
PROFESSOR AND DIRECTOR
DEPARTMENT OF INTERNAL MEDICINE
DVISION OF GENERAL CARDIOLOGY
UT SOUTHWESTERN MEDICAL CENTER
DALLAS, TX 75390

WEIR, MATTHEW R., MD *
PROFESSOR AND DIRECTOR
DIVISION OF NEPHROLOGY
DEPARTMENT OF MEDICINE
UNIVERSITY OF MARYLAND SCHOOL OF MEDICINE
BALTIMORE, MD 21201

WILLIAMS, JAMES KOUDY, DVM *
PROFESSOR
DEPARTMENT OF PATHOLOGY
SCHOOL OF MEDICINE
INSTITUTE FOR REGENERATIVE MEDICINE
WAKE FOREST UNIVERSITY
WINSTON-SALEM, NC 27101

ZHANG, MIN, PHD, MD *
PROFESSOR
DEPARTMENT OF STATISTICS
CENTER ON AGING AND THE LIFE COURSE
PURDUE UNIVERSITY
WEST LAFAYETTE, IN 47907

MAIL REVIEWER(S)

HOFFMAN, LUCAS R., PHD, MD
ASSOCIATE PROFESSOR
DIVISION OF PULMONARY AND SLEEP MEDICINE
DEPARTMENT OF PEDIATRICS
SEATTLE CHILDREN'S HOSPITAL
UNIVERSITY OF WASHINGTON
SEATTLE, WA 98105

SCIENTIFIC REVIEW OFFICER

WEBB, STEPHANIE JOHNSON, PHD
SCIENTIFIC REVIEW OFFICER
OFFICE OF SCIENTIFIC REVIEW/DERA
NATIONAL HEART, LUNG, AND BLOOD INSTITUTE
NATIONAL INSTITUTES OF HEALTH
BETHESDA, MD 20892

EXTRAMURAL SUPPORT ASSISTANT

MCCORKLE, ATHENA M., BS
EXTRAMURAL SUPPORT ASSISTANT
OFFICE OF SCIENTIFIC REVIEW/DERA
NATIONAL HEART, LUNG, AND BLOOD INSTITUTE
BETHESDA, MD 20892

PROGRAM REPRESENTATIVE

CAMPO, REBECCA A., PHD
PROGRAM DIRECTOR
DIVISION OF CARDIOVASCULAR SCIENCES
NATIONAL HEART, LUNG AND BLOOD INSTITUTE
BETHESDA, MD 20892

COADY, SEAN, MS
PROGRAM OFFICER
DIVISION OF CARDIOVASULAR SCIENCES
NATIONAL HEART, LUNG, AND BLOOD INSTITUTE
BETHESDA, MD 20892

EL KASSAR, NAHED, MD, PHD
MEDICAL OFFICER
DIVISION OF BLOOD DISEASES AND RESOURCES
NATIONAL HEART, LUNG, AND BLOOD INSTITUTE
NATIONAL INSTITUTES OF HEALTH
BETHESDA, MD 20892

HUANG, LI-SHIN, PHD
HEALTH SCIENTIST ADMINISTRATOR
DIVISION OF CARDIOVASCULAR SCIENCES
NATIONAL HEART, LUNG AND BLOOD INSTITUTE
BETHESDA, MD 20837

KALANTARI, ROYA, PHD
PROGRAM OFFICER
DIVISION OF LUNG DISEASE (DLD)
NATIONAL INSTITUTE OF HEART, LUNG AND BLOOD
BETHESDA, MD 20892

LAPOSKY, AARON, PHD
PROGRAM OFFICER
DIVISION OF LUNG DISEASES
NATIONAL HEART, LUNG, AND BLOOD INSTITUTE
BETHESDA, MD 20892

NATARAJAN, ARUNA R., MD, PHD
MEDICAL OFFICER
LUNG BIOLOGY AND DISEASE PROGRAM
NATIONAL HEART, LUNG AND BLOOD INSTITUTE
BETHESDA, MD 20892-7952

REDMOND, NICOLE MD, MD
MEDICAL OFFICER
CLINICAL APPLICATIONS AND PREVENTION BRANCH
NATIONAL HEART, LUNG AND BLOOD INSTITUTE
BETHESDA, MD 20817

REINECK, LORA A., MD
MEDICAL OFFICER
DIVISION OF LUNG DISEASES
NATIONAL HEART, LUNG, AND BLOOD INSTITUTE
BETHESDA, MD 20892

SARKAR, RITA, PHD
PROGRAM DIRECTOR
DIVISION OF BLOOD DISEASES AND RESOURCES
NATIONAL HEART, LUNG AND BLOOD INSTITUE
BETHESDA, MD 20814

SCOTT, JANE, SCD, MSN
PROGRAM OFFICIAL
OFFICE OF RESEARCH TRAINING AND CAREER DEVELOPMENT
OFFICE OF THE DIR, DIV OF CARDIOVASCULAR DISEASE
NATIONAL HEART, LUNG AND BLOOD INSTITUTE
BETHESDA, MD 20892

SMITH, SHARON M, PHD
PROGRAM OFFICER
TRANSLATIONAL BLOOD SCIENCE & RESOURCES BRANCH
NATIONAL HEART, LUNG, AND BLOOD INSTITUTE
NATIONAL INSTITUTES OF HEALTH
BETHESDA, MD 20892

TIGNO, XENIA, PHD
PROGRAM DIRECTOR
DIVISION OF LUNG DISEASES
NATIONAL HEART, LUNG AND BLOOD INSTITUTE
BETHESDA, MD 20892

WANG, WAYNE C., PHD
PROGRAM OFFICIAL
DIVISION OF CARDIOVASCULAR SCIENCES
NATIONAL HEART, LUNG, AND BLOOD INSTITUTE
BETHESDA, MD 20892

GRANTS MANAGEMENT REPRESENTATIVE

COBB, TARYN, MS
GRANTS MANAGEMENT SPECIALIST
OFFICE OF GRANTS MANAGEMENT
NATIONAL HEART, LUNG, AND BLOOD INSTITUTE
BETHESDA, MD 20892

OTHER REVIEW STAFF

MILES, LAUREN NICOLE, BS
STUDENT TRAINEE
NATIONAL INSTITUTES OF HEALTH
NATIONAL HEART, LUNG, AND BLOOD INSTITUTE
BETHESDA, MD 20892

* Temporary Member. For grant applications, temporary members may participate in the entire meeting or may review only selected applications as needed.

Consultants are required to absent themselves from the room during the review of any application if their presence would constitute or appear to constitute a conflict of interest.
